# Serum 25-hydroxyvitamin D levels are associated with carotid atherosclerosis in normotensive and euglycemic Chinese postmenopausal women: the Shanghai Changfeng study

**DOI:** 10.1186/1471-2261-14-197

**Published:** 2014-12-20

**Authors:** Hui Ma, Huandong Lin, Yu Hu, Xiaoming Li, Wanyuan He, Xuejuan Jin, Jian Gao, Naiqing Zhao, Zhenqi Liu, Xin Gao

**Affiliations:** Department of Geriatrics, Zhong Shan Hospital, Fudan University, Shanghai, 200032 China; Department of Endocrinology and Metabolism, Zhong Shan Hospital, Fudan University, Shanghai, 200032 China; Department of Ultrasonography, Zhongshan Hospital, Fudan University, Shanghai, 200032 China; Clinical Epidemiology Center, Zhong Shan Hospital, Fudan University, Shanghai, 200032 China; Department of Clinical Nutrition, Zhong Shan Hospital, Fudan University, Shanghai, 200032 China; Department of Biostatistics, College of Public Health, Fudan University, Shanghai, 200032 China; Division of Endocrinology and Metabolism, Department of Medicine, University of Virginia Health System, Charlottesville, Virginia USA

**Keywords:** 25-hydroxyvitamin D (25 (OH) D), Carotid intima-media thickness (CIMT), Carotid plaque, Carotid atherosclerosis

## Abstract

**Background:**

The role of serum 25-hydroxyvitamin D (25 (OH) D) in atherogenesis is unclear. We investigated whether the 25 (OH) D is independently associated with the carotid intima–media thickness (CIMT) and carotid plaques in normotensive and euglycemic postmenopausal women.

**Methods:**

A total of 671 normotensive and euglycemic postmenopausal women (mean age, 58.8 years) were enrolled from the Changfeng Study. A standard interview, anthropometrics measurements and laboratory analyses were performed for each participant. Bilateral CIMTs were measured using ultrasonography, and the presence of carotid plaques was assessed. The serum 25 (OH) D was measured using electrochemiluminescence immunoassay.

**Results:**

Serum 25 (OH) D was 43.6 ± 18.2 nmol/L in the postmenopausal women. Compared with subjects with 25 (OH) D in the first, second and third quartiles, subjects with 25 (OH) D in the fourth quartile had decreased CIMT and prevalence of carotid plaque (0.684 ± 0.009 mm vs 0.719 ± 0.009 mm, 0.708 ± 0.009 mm and 0.709 ± 0.009 mm; 10.8% vs 19.0%, 14.8% and 16.8%, respectively). After adjusting for conventional CVD risk factors, PTH, liver and renal function, postmenopausal women with 25 (OH) D in the fourth quartile still had lower CIMT than those in the first, second and third quartiles (p = 0.039) and the subjects in the fourth quartile had a 0.421-fold decreased risk of carotid plaques relative to those in the lowest quartile (95% confidence interval 0.209 to 0.848).

**Conclusions:**

These results suggest serum 25 (OH) D is independently and inversely associated with carotid atherosclerosis in postmenopausal women with normal blood pressure and normal glucose tolerance.

## Background

Vitamin D, a fat-soluble vitamin and steroid hormone, is well known for its pivotal role in calcium homeostasis and bone metabolism. Beyond the role in bone health, vitamin D is receiving increasing attention for its influence on non-skeletal health problems and chronic diseases [1, 2]. Serum 25-hydroxyvitamin D (25(OH) D), the major storage form of vitamin D, is formed in the liver, and is a clinical indicator of overall vitamin D3 status [3]. The association of serum 25 (OH) D levels with cardiovascular diseases (CVD) is debated in the literature. The Framingham Offspring Study revealed that low serum 25 (OH) D was independently associated with an increased incidence of CVD in Caucasians during a 5.4-year follow-up period [4]. Additionally, European studies have shown that serum 25 (OH) D levels are inversely associated with the prevalence of CVD and carotid intima-medial thickening in patients with type 2 diabetes mellitus (DM) [5]. More recently, the meta-analysis by Wang et al. [6] of nineteen independent prospective cohort studies reported an inverse association between serum 25 (OH) D concentrations and risk of CVD outcome. However, several other studies observed no significant association between serum 25 (OH) D levels and CVD. For example, Deleskog A et al and Blondon M et al demonstrated that levels of 25 (OH) D showed multiple associations with established and emerging cardiovascular risk factors but were not consistently, independently related to measures of carotid IMT and carotid plaque [7, 8]. The Korean National Health and Nutrition Examination Survey (KNHANES-2008-2009) indicates that the prevalence of CVD is not associated with low serum 25 (OH) D levels in the total population [9]. The study by Melamed et al. [10], using the third National Health and Nutrition Examination Survey cohort, while CVD risk was not statistically significant, the all-cause mortality data suggested a U- or reverse J-shaped dose-relationship, with increased total mortality for both the lowest and highest serum 25 (OH) D concentrations (<44.4 and >80.1 nmol/l, respectively) in this cohort followed for 9 years. As noted, the majority of the previous studies were conducted using populations with different proportions of hypertension or diabetes. In addition, 25 (OH) D is associated with hyperglycemia and hypertension [11], which may themselves be linked to CVD. Therefore, the association between 25 (OH) D and atherosclerosis may be confounded by the inclusion of subjects with hypertension, diabetes or pre-diabetes. Moreover, it has been shown that 25 (OH) D values vary between different ethnicities. There are few reports on the relationship between 25 (OH) D and CVD in the Chinese postmenopausal women. Given the high risk of CVD in this population [12], more evidence is needed to evaluate the association between 25 (OH) D and atherosclerosis in the Chinese postmenopausal women.

The use of high-resolution color-coded duplex sonography offers the opportunity to assess the carotid intima-media thickness (CIMT) and carotid plaques as reliable markers of atherosclerosis [13]. Therefore, in the present study, we investigated the relationship between 25 (OH) D and carotid atherosclerosis in a community-based population of normotensive and euglycemic postmenopausal women.

## Methods

### Study population

The subjects were participants in the Changfeng Study, a community-based study of chronic diseases among middle-aged and elderly individuals which has been described elsewhere [14]. The Changfeng community is a middle-class community in Shanghai [14]. From June 2009 to June 2012, 2717 postmenopausal women were initially enrolled. We excluded 2046 participants for the following reasons: lack of physical examination and laboratory assessments (n = 51), prevalent CVD (myocardial infarction, stroke, or peripheral arterial disease) (n = 202), prevalent hemodialysis (n = 2), bone fracture within 3 months and use of drugs known to influence bone metabolism including the use of postmenopausal hormone therapy, calcium, diphosphonate, vitamin D and glucocorticoid (n = 384), prevalent hypertension (systolic blood pressure ≥ 140 mmHg, diastolic blood pressure ≥90 mmHg, the use of antihypertensive medications, or diagnosed hypertension) (n = 1076), prevalent diabetes mellitus or pre-diabetes (fasting glucose ≥5.6 mmol/L, OGTT 2 h glucose ≥7.8 mmol/L, the use of hypoglycaemic medications, or diagnosed diabetes; n = 320), or the use of lipid-lowering therapy or use of the antiplatelet agents (n = 11). Finally, 671 subjects were included in the analysis.

The study was approved by the ethical committee of Zhongshan Hospital, Fudan University and was conducted in accordance with the guidelines of the Declaration of Helsinki. All the patients provided consent upon enrolment in the study. Interviews, physical examinations and ultrasound scans were performed at the Changfeng Community Health Service Center.

### Clinical measurements

Letters were sent to participants with instructions asking them not to alter their diet or level of physical activity for at least 3 days before the test. A questionnaire was administered by trained nurses to evaluate the medical history and lifestyle of each participant. Weight and height were measured while the participant was clothed in a light gown. The body mass index (BMI) was calculated as the weight divided by the height squared (kg/m^2^). The waist circumference was measured midway between the lowest rib margin and the iliac crest in a standing position, and the hip circumference was measured at the widest level over the greater trochanters. The waist-to-hip ratio (WHR) was calculated as the waist circumference divided by the hip circumference. The resting blood pressure was measured three times, and the mean value was used for the analysis. Blood samples were obtained after a fasting period of at least 10 hours. Total cholesterol (TC), high-density lipoprotein cholesterol (HDL-C), triglycerides (TG) and liver enzymes were measured using a model 7600 automated bio-analyser (Hitachi, Tokyo, Japan). The level of low-density lipoprotein cholesterol (LDL-C) was calculated using the Friedewald equation. The fasting blood glucose (FBG) and 2 h glucose levels following a 75-g oral glucose challenge (PPG) for non-diabetics were measured using the glucose oxidase method. The glomerular filtration rate (GFR) was estimated based on the serum creatinine concentration using the Modification of Diet in Renal Disease (MDRD) formula: estimated GFR (eGFR) = 186 × [serum creatinine (mmol/L) × 0.0113]^- 1.154^ × age^-0.203^ × (0.742 for women) [15]. The serum 25(OH)D, parathyroid hormone (PTH) and insulin were measured by electrochemiluminescence immunoassay using an immunoassay analyzer (Roche Cobas-6001, Switzerland; coefficient of variation <4.0%, <5.0% and <5.0%, respectively). Homeostasis model assessment index for insulin resistance (HOMA-IR) and beta cell function (HOMA-%B) were used to estimate insulin sensitivity and insulin secretion [16].

The carotid arteries of the participants were evaluated by an experienced radiologist who was blinded to the participants’ details using a GE Logic P5 (GE Healthcare, Milwaukee, USA) scanner with a 10-MHz probe. The CIMTs on both sides were measured in the common carotid artery approximately 1 cm proximal to the bifurcation at the far wall during end diastole. The CIMT was quantified at plaque-free sections of the carotid arteries as the distance between the lumen–intima and media–adventitia interfaces. Three values were measured on each side, and the average CIMT values were used for the analysis. The study procedure involved scanning the near and far walls of both common carotid arteries, the carotid bifurcation, and the internal carotid artery for the presence of plaques, defined as the presence of focal wall thickening resulting in a thickness that is at least 50% greater than that of the surrounding vessel wall or as a focal region with a CIMT greater than 1.5 mm that protrudes into the lumen that is distinct from the adjacent boundary, according to American Society of Echocardiography [17]. Repeated measurements on the same subjects (performed in 104 subjects) for CIMT and carotid plaque yielded an intraclass correlation coefficient (ICC) of 95% (95% confidence interval, 0.91 to 0.97) and 96% (95% confidence interval, 0.92 to 0.97), respectively.

Hypertension was defined according to the Seventh Report of the Joint National Committee [18]. The diagnoses of impaired fasting glucose, impaired glucose tolerance, and DM were based on the American Diabetes Association 2010 criteria [19]. The diagnosis of cardiovascular disease was based on self-reports and confirmed using hospital medical records.

### Statistical analyses

The data were expressed as the means ± SD(SE), frequencies or medians with 25th and 75th percentiles. Skewed variables were logarithmically transformed to improve normality prior to analysis. To evaluate the relationship between each parameter and the 25(OH)D, the subjects were stratified according to the 25(OH)D quartiles. The ranges of 25(OH)D in the quartiles were 12.4-30.7, 30.8 -40.5, 40.6-53.0 and 53.2-153.0 nmol/L. Analysis of covariance and logistic regression, with adjustments for CVD risk factors, liver enzymes and the GFR were conducted to compare means and proportions, respectively, across the 25(OH)D quartiles. We used a general linear model analyses and complete the homogeneity tests, in which the outcome is the CIMT value of comparison among 25(OH)D quartiles. We test the null hypothesis that the error variance of the dependent variable is equal across groups and get the result that p = 0.366. Regression coefficients and odds ratios (ORs) were calculated for a 1-unit increase in 25(OH)D. SPSS 16.0 for Windows (SPSS 16.0 Inc Chicago, IL, USA)) was used to perform the statistical analyses. All statistical tests were two tailed, and p-values less than 0.05 were considered significant.

## Results

### Characteristics of the subjects according to the 25(OH)D quartiles

A total of 671 postmenopausal women were evaluated. The mean value of CIMT was 0.703 ± 0.123 mm. The prevalence of carotid plaques was 15.4%. The mean value of 25(OH)D was 43.6 ± 9.2 nmol/L. A total of 1.5% of the subjects was current smokers. Table 1 shows the clinical and biochemical parameters according to the quartiles groups for the 25(OH)D. When the traditional CVD risk factors were examined, WHR, DBP, PPG and PTH were significantly associated with the 25(OH)D quartiles. Other parameters were not significantly different among the groups.

**Table 1:** **Characteristics of the subjects according to quartiles groups for 25**(**OH**)**D in subjects**

Variables	All (n = 671)	1 ^st^ quartile (n = 168)	2 ^nd^ quartile (n = 169)	3 ^rd^ quartile (n = 167)	4 ^th^ quartile (n = 167)	P among groups
25(OH) D range (nmol/L)	12.4-153.0	12.4-30.7	30.8 -40.5	40.6-53.0	53.2-153.0	
Age (ys)	58.8(7.5)	57.9(7.4)	59.5(7.5)a	58.9(7.0)	59.0(8.0)	0.255
BMI (kg/m^2^)	22.8(2.9)	22.9(2.9)	22.8(2.8)	23.1(3.1)	22.4(2.8)c	0.238
WHR	0.853(0.063)	0.846(0.052)	0.855(0.061)	0.847 (0.061)	0.863(0.074)a,c	0.042
Current smoker, n (%)	10(1.5%)	4(2.4%)	3(1.8%)	1(0.6%)	2(1.2%)	0.572
SBP (mmHg)	120.0(11.1)	119.7(11.8)	120.8(11.1)	120.8(10.3)	118.7(11.1)	0.253
DBP (mmHg)	70.4(7.5)	71.2(7.6)	70.0(7.2)	71.3(7.3)	69.1(7.8)a,c	0.023
ALT (U/L)	16.9(9.5)	15.6(7.6)	16.4(7.2)	17.6(7.1)	17.8(7.5)a	0.107
AST (U/L)	21.1(6.1)	20.5(6.9)	20.7(5.5)	21.4(5.5)	21.7(6.5)	0.246
TC (mmol/L)	5.2(0.8)	5.2(0.8)	5.3(0.9)	5.4(0.9)	5.2(0.7)c	0.130
LDL-c (mmol/L)	3.0(0.7)	3.0(0.8)	3.0(0.7)	3.1(0.8)	2.9(0.7)c	0.096
HDL-c (mmol/L)	1.6(0.4)	1.6(0.4)	1.6(0.4)	1.6(0.4)	1.6(0.4)	0.605
TG (mmol/L)	1.4(0.7)	1.3(0.6)	1.3(0.6)	1.4(0.8)	1.4(0.7)	0.467
FBG (mmol/L)	4.9(0.3)	4.9(0.3)	4.9(0.4)	4.9(0.3)	4.9(0.3)	0.516
PPG (mmol/L)	5.7(1.1)	5.9(1.1)	5.5(1.0)a	5.4(1.0)a	5.4(0.9)a	<0.001
PTH (pg/mL)	42.2(15.1)	48.6(16.3)	42.9(13.6)a	41.4(14.5)a	35.7(12.9)a,b,c	<0.001
HOMA-IR	1.4(1.0-1.9)	1.3(1.0-2.0)	1.5(1.1-2.0)	1.5(1.0-2.0)	1.4(1.0-1.8)	0.425
HOMA-B %	97.4(72.7-128.6)	92.1(71.4-127.8)	98.6(75.6-131.0)a	102.1(70.8-134.7)	97.5(73.4-122.9)	0.1
GFR (ml/min per 1.73 m^2^)	96.9(18.9)	98.3(19.0)	96.2(19.2)	97.4(19.8)	96.0(17.6)	0.647

Data are mean (SD) or percentage of subjects or median (interquartile range).

BMI: body mass index, WHR: waist-hip-ratio, FBG: Fasting blood glucose, PPG: OGTT 2 h blood glucose, PTH: parathyroid hormone, SBP: systolic blood pressure, DBP: diastolic blood pressure, ALT: alanine transaminase, AST: aspartate transaminase, TC: Total cholesterol, TG: triglyceride, HDL-C: high density lipoprotein cholesterol, LDL-C: low density lipoprotein cholesterol, HOMA-IR: homeostasis model assessment index for insulin resistance, HOMA-B: homeostasis model assessment index for beta cell function, GFR: glomerular filtration rate.

a Analysis of variance with LSD post-hoc test or Chi-square statistical analysis: P < 0.05 versus 1st quartile.

b Analysis of variance with LSD post-hoc test or Chi-square statistical analysis: P < 0.05 versus 2nd quartile.

c Analysis of variance with LSD post-hoc test or Chi-square statistical analysis: P < 0.05 versus 3rd quartile.

### Association of the anthropometric and biochemical parameters with serum 25(OH)D levels

Linear regression analysis showed an association between 25(OH)D and BMI, WHR, DBP, PPG and PTH (Table 2). Multivariate linear stepwise regression analysis was performed to evaluate the independent factors of 25(OH)D. The analysis demonstrated that age (standardized β = 0.082, p = 0.028), PTH (standardized β = -0.334, p < 0.001), BMI (standardized β = -0.141, p = 0.001) and PPG (standardized β = -0.053, p = 0.003) were independently associated with serum 25(OH)D.

**Table 2:** **Association of the anthropometric and biochemical parameters with serum 25**(**OH**)**D levels in postmenopausal women**

	β (95% CI)	Standardized β	P
Age (per 1y)	0.154(-0.029-0.338)	0.064	0.1
BMI (per 1 units)	-0.593(-1.063- -0.123)	-0.095	0.013
WHR	25.129(3.130-47.128)	0.086	0.025
Current smoking	-5.456(-16.840-5.928)	-0.036	0.347
SBP (per 1 mmHg)	-0.104(-0.229-0.020)	-0.064	0.1
DBP (per 1 mmHg)	-0.272(-0.455- -0.09)	-0.112	0.004
ALT (1U/L)	0.130(-0.014-0.275)	0.068	0.077
AST (1U/L)	0.161(-0.064-0.386)	0.054	0.161
TC (1 mmol/L)	-0.679(-2.323-0.965)	-0.031	0.418
LDL-C (1 mmol/L)	-1.199(-3.044-0.646)	-0.049	0.202
HDL-C (1 mmol/L)	-1.489(-5.121-2.143)	-0.031	0.421
TG (1 mmol/L)	-1.838(-2.788-0.111)	-0.071	0.065
FBG (1 mmol/L)	-2.853(-6.953-1.247)	-0.053	0.172
PPG (1 mmol/L)	-2.569(-3.862- -1.275)	-0.149	0.001
PTH (1 pg/mL)	-0.408(-0.494- -0.322)	-0.338	<0.001
logHOMA-IR	-4.853(-11.222-1.516)	-0.058	0.135
logHOMA-B %	1.779(-0.079-3.480)	0.025	0.125
GFR (1 ml/min per 1.73 m2)	-0.052(-0.125-0.021)	-0.054	0.161

*BMI*: body mass index, *WHR*: waist-hip-ratio, *FBG*: Fasting blood glucose, *PPG*: OGTT 2 h blood glucose, *PTH*: parathyroid hormone, *SBP*: systolic blood pressure, *DBP*: diastolic blood pressure, *ALT*: alanine transaminase, *AST*: aspartate transaminase, *TC*: Total cholesterol, *TG*: triglyceride, *HDL*-*C*: high density lipoprotein cholesterol, *LDL*-*C*: low density lipoprotein cholesterol, *HOMA*-*IR*: homeostasis model assessment index for insulin resistance, *HOMA*-*B*: homeostasis model assessment index for beta cell function, *GFR*: glomerular filtration rate.

### Association between carotid atherosclerosis and 25(OH)D

**Figure 1:**
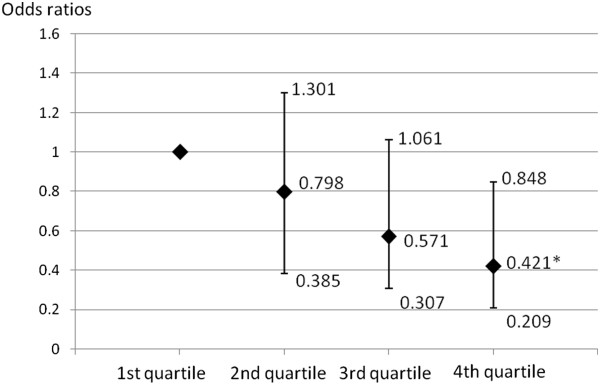
**ORs of carotid plaque in the subjects across the 25** **(OH)** **D quartile.** ORs of carotid plaque in the subjects according to 25(OH)D quartile groups after adjusting for age, BMI, WHR, FBG, PPG, SBP, DBP, TG, HDL-C, LDL-C, smoking, ALT, AST, PTH, logHOMA-IR, logHOMA-B% and the GFR, respectively. * Analysis of variance with logistic regression analysis: P < 0.05 versus 1st quartile ORs and 95% CI are shown.

Table 3 presents the CIMT of the subjects according to 25(OH)D quartile groups. Compared with the subjects in the first, second and third 25(OH)D quartiles, those in the fourth quartile had significantly thinner CIMTs (0.684 ± 0.009 mm vs 0.719 ± 0.009 mm, 0.708 ± 0.009 mm and 0.709 ± 0.009 mm, respectively). After adjusting for conventional CVD risk factors, liver enzymes and the GFR, the subjects with 25(OH)D in the fourth quartile still had lower CIMT than those in the first, second and third quartiles (p = 0.039). Compared with the subjects in the first, second and third 25(OH)D quartiles, those in the fourth quartile had significantly lower prevalence of carotid plaque (10.8% vs 19.0%, 14.8% and 16.8%, respectively). After adjusting for conventional CVD risk factors, liver enzymes and the GFR, the subjects with 25(OH)D in the fourth quartile had 0.421-fold decreased risks for carotid plaques relative to those in the lowest quartile (95% confidence interval 0.209 to 0.848) (Figure 1).

**Table 3:** **CIMT in the subjects according to quartile groups for 25**(**OH**)**D in the subjects**

	Quartile groups for 25(OH) D in the subjects				
	1 ^st^ quartile	2 ^nd^ quartile	3 ^rd^ quartile	4 ^th^ quartile	p
Unadjusted					
CIMT (mm)	0.719(0.009)	0.708(0.009)	0.709(0.009)	0.684(0.009)a,b,c	0.034
Model I					
CIMT (mm)	0.712(0.009)	0.704(0.009)	0.707(0.009)	0.688(0.009)a,b,c	0.039

Data are mean (SE).

a Analysis of variance with LSD (least significant difference) post-hoc test: P < 0.05 versus 1st quartile.

b Analysis of variance with LSD post-hoc test: P < 0.05 versus 2nd quartile.

c Analysis of variance with LSD post-hoc test: P < 0.05 versus 3rd quartile.

Model I: adjusting for age, *FBG* (fasting blood glucose), *PPG* (postprandial blood glucose), PTH(parathyroid hormone), *BMI* (body mass index), *WHR* (waist-to-hip ratio), current smoking, SBP (systolic blood pressure), *DBP* (diastolic blood pressure), *ALT*(alanine transaminase), *AST*(aspartate transaminase),*TG* (triglyceride), *HDL*-*C* (high-density lipoprotein cholesterol), *LDL*-*C* (low-density lipoprotein cholesterol), logHOMA-IR (homeostasis model assessment index for insulin resistance), logHOMA-%B (homeostasis model assessment index for beta cell function) and *GFR*(glomerular filtration rate).

cIMT: carotid intima-media thickness, 25 (OH) D: 25-hydroxyvitamin D.

## Discussion

Our study showed that serum 25(OH)D was independently associated with carotid atherosclerosis in normotensive and euglycemic Chinese postmenopausal women. The CIMT and the prevalence of carotid plaques significantly decreased with increasing serum 25(OH)D levels after adjusting for conventional CVD risk factors, PTH, liver enzymes and renal function in the subjects.

Serum 25(OH)D has recently become an interesting topic in cardiovascular researches [1–6]. Although a number of studies have demonstrated that 25(OH)D is associated with the development of cardiovascular events, it is still conflicting whether there is an independent association between 25(OH)D and atherosclerotic CVD. The participants in the Framingham Offspring Study [4] had 40% patients with hypertension and 8% diabetic patients. European studies [5] were performed in diabetic adults. The studies eligible for inclusion in the meta-analysis by Wang [6], the study by Deleskog A [7], Blondon M [8], Melamed [10] and the Korean National Health and Nutrition Examination Survey [9] enrolled study populations with different proportions of hypertension or diabetes. As noted, recent studies have demonstrated that there was a close association between 25(OH)D and cardiovascular risk factors, such as hyperglycemia and hypertension [11]. Studies suggest vitamin D deficiency may be a contributor to the development of CVD potentially through associations with diabetes or hypertension [20]. In line with previous studies [11], serum 25(OH)D concentrations were inversely associated with blood pressure and blood glucose in the present study. Therefore, the relationship between carotid atherosclerosis and serum 25(OH)D would be affected by the chronic effects of increased blood glucose levels and blood pressure. The impact of residual confounding factors would remain after adjusting for hyperglycemia and hypertension in previous studies. To eliminate the confounding effects of hyperglycemia and hypertension on the relationship between carotid atherosclerosis and serum 25(OH)D, we explored the relationship in the subjects with normal blood pressure and normal glucose tolerance. Thus, the association between 25(OH)D and carotid atherosclerosis was not confounded by hyperglycemia and hypertension.

In the present study there was an independent association between 25(OH)D and CIMT after adjusting for established cardiovascular risk factors. As we known, CIMT may be affected both by atherosclerosis and wall hypertrophy, we also assessed the association between 25(OH)D and carotid plaque, which may be more representative of atherosclerosis than CIMT and more informative for predicting cardiovascular risk [21]. We demonstrated a negative and independent relationship between 25(OH)D and carotid plaque, independently from the traditional risk factors. In contrast, Deleskog A et al [7] reported that there were no independent relationships between 25(OH)D and the baseline and progression measures of carotid IMT in 3430 middle-aged and elderly subjects with high cardiovascular risk but no prevalent CVD. Similarly, Blondon M et al [8] evaluated the associations of 25(OH)D with CIMT and carotid plaques among 3251 participants free of cardiovascular disease in the Multi-Ethnic Study of Atherosclerosis and observed consistent null results for both cross-sectional associations and longitudinal associations evaluating change in IMT and incident plaque during 10 years of follow-up. Several explanations could be involved in the above different results. First, the BMI, blood pressure, blood glucose and lipid profile of the subjects in our study were relatively improved in comparison with those in the above studies. In addition, our subjects had a lower proportion of smoking participants. Thus, the association between 25(OH)D and CVD in subjects with higher cardiovascular risk may be weakened for the greater contribution of traditional atherosclerotic risks to CVD in the studies of Deleskog A [7] and Blondon M [8]. Second, there may be sex-related differences in the association between 25(OH)D and CVD. These sex-related differences might depend on background data. It is known that the life-long risk for CVD is higher for men than women [22]. Third, it is necessary to take ethnic differences into account.

69.9% of subjects have 25(OH)D levels below 50 nmol/L. Similarly, another study in China evaluated vitamin D status of healthy adults living in Guiyang (latitude 26.5° north). The study showed that the average serum 25(OH)D level of 20.4 ng/mL(51 nmol/L) and serum 25(OH)D was below 50 nmol/L in 52.3% [23]. Lu L et al [24] measured plasma 25(OH)D was in a cross-sectional sample of 1,443 men and 1,819 women aged 50-70 years from Beijing (latitude 40° north) and Shanghai (latitude 31° north). The median value of plasma 25(OH)D was 35.6 nmol/l in Beijing and 47.6 nmol/l in Shanghai, and the percentages of vitamin D deficiency, insufficiency, and sufficiency were 69.2, 24.4, and 6.4%, respectively. Indeed, poor vitamin D status in middle and older Chinese individuals was also reported previously in two small bone related studies conducted in Beijing [25] and Shenyang [26]. The above data suggested that vitamin D deficiency was common in middle-aged and elderly Chinese individuals. Another explanation is the exclusion criteria of the intake of the vitamin D supplements in our study. Additionally, unlike in the United States and other western countries, a racial/ethnic difference may be exist in the levels of 25(OH)D concentrations. Although little is known regarding to the high prevalence of vitamin D deficiency in our population, the criteria of vitamin D deficiency in Chinese may differ from that in the western population.

The mechanisms responsible for the independent relationship between 25(OH)D and atherosclerosis have not been well elucidated. In our study, 25(OH)D was negatively associated with BMI, DBP and PPG. These findings were also supported by other studies [27–29]. Therefore, the beneficial effects of 25(OH)D on atherosclerosis might be attributed to its ability to improve the glucose metabolism and blood pressure control. On the other hand, in the present study, we demonstrated an inversely and independent association between 25(OH)D and carotid atherosclerosis after adjustment for established CVD risk factors, PTH, liver and renal function in postmenopausal women with normal blood pressure and normal glucose tolerance. If 25(OH)D and the other risk factors share a common causal pathway, adjusting for these risk factors may attenuate the relationship between 25(OH)D and carotid atherosclerosis. However, 25(OH)D remained a relatively strong predictor after full adjustment in our study, suggesting that there was an independent additive component in the relationship between 25(OH)D and CVD. Thus, other atherogenic mechanisms could conceivably be involved. Because many cell types involved in cardiovascular function like cardiomyocytes, endothelial cells, or vascular smooth muscle cells express vitamin D receptors, a direct influence of vitamin D on the cardiovascular system can be assumed [30]. There are several mechanisms proposed to explain the inverse relationship between vitamin D and CVD. First, activated vitamin D is an inhibitor of the renin-angiotensin system [31]. Vitamin D deficiency predisposes to up-regulation of the renin–angiotensin–aldosterone system and hypertrophy of the vascular smooth muscle cells [20, 32]. Second, Vitamin D has effects that may favorably influence cardiovascular system through strengthen in insulin secretion and insulin sensitivity [33], down-regulate coagulation through the up-regulation of thrombomodulin [34], down-regulate vascular calcification [35–37] and modulation of inflammatory processes [38]. Third, long-term vitamin D insufficiency and deficiency cause secondary hyperparathyroidism, which in turn may mediate many of the detrimental CV effects including increasing systemic inflammation, as indicated by increased levels of C-reactive protein, homocysteine, and interleukin-10 [32]. Fourth, vitamin D may play a pivotal role in cardiac function. Cardiac muscle cells possess a vitamin D receptor and a 1,25-dihydroxyvitaminD–dependent calcium-binding protein. Vitamin D has effects on extra- cellular matrix remodeling, myocardial cell hypertrophy, and proliferation [20, 39]. Vitamin D deficiency predisposes to lead to hypertrophy of the left ventricle. Fifth, activated vitamin D may also retard atherosclerosis by inhibiting macrophage cholesterol uptake and foam cell formation [40].

Our results suggest that 25(OH)D is a marker or risk factor for atherosclerosis. 25(OH)D could be adopted as an additional marker of the detection of CVD and the implementation of interventions. An early evaluation of the 25(OH)D would be advantageous for the early detection of CVD, and individuals with decreased 25(OH)D might benefit from more aggressive lifestyle modifications and food-based strategies.

We recognize several limitations of this study: it is cross-sectional, and the 25(OH)D was assessed based on a single morning fasting blood sample. We also acknowledge that in this analysis we do not have data on hours of sunlight per day, albumin and vitamin D binding protein. The cross-sectional nature of this study limits our ability to determine causality. The potential confounding factors remain, particularly in the absence of data for physical activity and socio-economic status. However, the temporal relationship between the 25(OH)D and carotid atherosclerosis has been well established. Our participants were postmenopausal women, and therefore, the results cannot be applied to younger subjects. Hence, the association between 25(OH)D and carotid atherosclerosis should be confirmed using a larger sample and in prospective studies.

## Conclusions

In conclusion, we demonstrated that 25(OH)D has a negatively correlation with carotid atherosclerosis even after adjusting for conventional CVD risk factors, PTH, liver and renal function in postmenopausal women with normal blood glucose levels and normal blood pressure. Our findings suggest that individuals with decreased 25(OH)D require aggressive management of CVD risk factors. Should causality be affirmed by ongoing and future studies, there are food-based strategies for enhanced vitamin D status in the population which could ultimately lower risk of CVD.

## Abbreviations

25(OH)D: 25-hydroxyvitamin D
CIMT: Carotid intima-media thickness
CVD: Cardiovascular disease
BMI: Body mass index
WHR: Waist-hip-ratio
FBG: Fasting blood glucose
PPG: OGTT 2 h blood glucose
PTH: Parathyroid hormone
SBP: Systolic blood pressure
DBP: Diastolic blood pressure
ALT: Alanine transaminase
AST: Aspartate transaminase
TC: Total cholesterol
TG: Triglyceride
HDL-C: High density lipoprotein cholesterol
LDL-C: Low density lipoprotein cholesterol
HOMA-IR: Homeostasis model assessment index for insulin resistance
HOMA-B: Homeostasis model assessment index for beta cell function
GFR: Glomerular filtration rate.
